# Pathology of Tuberculosis

**Published:** 1915-07

**Authors:** E. C. Thrash

**Affiliations:** Atlanta, Ga.


### Journal-Record of Medicine

Successor to Atlanta Medical and Surgical Journal, Established 1855
and Southern Medical Record, Established 1870

**OWNED BY THE ATLANTA MEDICAL JOURNAL COMPANY**

PUBLISHED MONTHLY

*Official Organ Fulton County Medical Society, State Examing Board, Atlanta, Birmingham and Atlantic Railroad Surgeon's Association, Chattahoochee Valley Medical and Surgical Association, Etc.*

RICHARD R. DALY, M. D., Editor
BERNARD WOLFF, M. D., Supervising Editor
A. W. STIRLING, M. D., C. M., D. P. H.; J. S. HURT, B. Ph., M.D.
GEO. M. NILES, M. D., W. J. LOVE, M. D., (Ala.); Associate Editors
E. W. ALLEN, Business Manager

**COLLABORATORS**

W. F. WESTMORELAND, M. D., General Surgery
F. W. McRAE, M. D., Abdominal Surgery
ALLEN H. BUNCE, Medical Societies
E. B. BLOCK, M. D., Diseases of the Nervous System
MICHAEL HOKE, M. D., Orthopedic Surgery
CYRUS W. STRICKLER, M. D., Legal Medicine and Medical Legislation
E. C. DAVIS, A. B., M. D., Obstetrics
E. G. JONES, A. B., M. D., Gynecology
ALLEN H. BUNCE, M. D., Pathology and Bacteriology
R. T. DORSEY, JR., B. S., M. D., Medicine
L. M. GAINES, A. B., M. D., Internal Medicine
GEO. C. MIZELL, M. D., Diseases of the Stomach and Intestines
L. B. CLARKE, M. D., Pediatrics
EDGAR PAULLIN, M. D., Opsonic Medicine
THEODORE TOEPEL, M. D., Mechano Therapy
R. R. DALY, M. D., Etc., Diseases of the Eye, Ear, Nose and Throat
BERNARD WOLFF, M. D., Diseases of the Skin
E. G. BALLENGER, M. D., Diseases of the Genito-Urinary Organs

VOL. LXII. ATLANTA, GA., JULY, 1915. NO. 4

### PATHOLOGY OF TUBERCULOSIS.

E. C. THRASH, M. D., ATLANTA, GA.

The pathology of tuberculosis is characterized chiefly by a deposit of tubercles in the tissues, resulting from the reaction of these tissues to the invasion of tubercle bacilli. This reaction is confined in the beginning principally to fixed cells, the chief of which are derived from surrounding connective tissue and endothelium of capillary vessels. Paranchymatous cells, however, in glandular organs such as the liver are often found engaged in these activities.

Tubercle bacilli find entrance into the body through the respiratory and digestive tracts and occasionally through the skin. The latter mode is so rare that we shall dismiss it simply by stating that after long exposure of the skin to tubercle bacilli they find lodgment in the pores and in due process of time develop tubercles. Systemic tuberculosis is almost invariably produced by either the ingestion or inhalation of the germs. Experience and experiments have caused me to conclude that they usually enter through the digestive tract.

It has been proven that the defensive products of the somatic cells will not permit of multiplication of tubercle bacilli from a less number of germs than ten or fifteen massed together, and for this to occur at all there must be a resting period of two or three weeks, during which time they must be undisturbed in order that growth may take place. In the meantime there begins a proliferation of cells surrounding these bacilli, which, stimulated by the germs, produce an incipient tubercle. This is the nidus in which these germs begin to propagate, and produce their dire and deadly results.

This process of entrance takes place as follows: Tubercle bacilli find their way into adjacent lymphatic glands through the follicles and crypts of the tonsils, broken mucous membrane in the mouth and especially around the teeth, follicles and crypts in the pharynx, mucous gland follicles in the bronchi, and villi of the intestines. The phagocytes, no doubt, play an important role in this process by picking out tubercle bacilli from the follicles and crypts of the mucous membrane and being unable to destroy them, ultimately land together with their refractory germs into lymphatic glands where their fate is similar to that of the ones described later, which enter the glands through the intestines. Those which enter the stomach and survive the ravages of the gastric juice pass into the lymphatic glands from the intestines through the villi after being incorporated in emulsified fats.

When these germs are deposited in the various glands by phagocytic action or through emulsified fats they are either disintegrated by the cellular structures of the glands which is the usual course, or exceptionally they are massed in these glands to a degree that infection takes place. Later nature may show a resistance, stop the progress of germ development and restore the glands to normal. On the other hand the process may continue until there is a caseation, softening, degeneration and breaking down of these glands. There being a constant flow of lymph through these structures toward the blood stream, the germs find their way into the circulation of the blood either singly, in clumps, or in the debris of broken down glands. The single ones are whipped around in the blood until they are ultimately destroyed. The clumps and debris are either broken to pieces and meet the fate of the single ones, or move on until they find a vessel through which they cannot pass.

Since this material enters the blood stream through the veins, the first capillary obstruction which it meets is in the lungs. The elasticity of the capillary system of the lungs in children usually allows the tuberculous masses to pass through to the systemic circulation, and they lodge in the more constricted vessels of the bony structures, hence the great number of joint and bone tuberculosis in children. When these clumps of tubercle bacilli plug up capillaries or arterioles, either in the lungs or other organs with terminal circulation, the result is death of the microscopic area that these respective pessels supply. This microscopic infarct supplies pabulum for the germs and inhibits invasion of antibodies until the germs have time to get a foothold, and multiply sufficiently to protect themselves against somatic cell activities. One who makes a careful study of tuberculosis cannot help but arrive at this conclusion.

The development of a tubercle is now under way. The poisonous bacilli, together with the necrosed cells that surround them, serve as a stimulus for proliferation of surrounding connective tissue cells. Mitosis in these begins to take place more or less actively, the results of which are fibroblasts.

These fibroblasts are motile and form a protective wall around the enemy. The capillary vessels are destroyed primarily by being plugged, and later by being pressed upon from cell proliferation and poisoned by bacterial toxines. This causes the endothelial structures of the vessels to change from flattened cells for the purpose of conveying blood to round defensive ones, which assume the same protective role as the fibroblasts do. These are now called epithelioid cells. All vessels are destroyed by this process as they are encroached upon, hence the lack of blood supply in a tubercle. Migration now of lymphoid cells begins to take place. There are but few polymorphonuclear leukocytes in the tubercle on account of their partial inability to digest and destroy tubercle bacilli, therefore, nature builds bulwarks instead of using bayonets.

The massing of the cells in this manner without blood supply and with poisons weakening and destroying them at the center where the germs are active causes cell death to begin at this point. The disintegrated cells, together with bacteria and their metabolic products first form a coagulation necrosis. Material in the form of fibroblasts, epithelioid cells and lymphocytes from the periphery, continues to be laid down to thicken the protective wall, while it is continuously weakening and dying at the center. The result of this death, on account of the fat contained in the tubercle and the fatty degeneration taking place in the necrobiosis of the cells, is a white cheesy mass, caseation.

Where there are a great number of these tubercles developing near each other the natural result is for these caseous areas to merge until the mass gets sufficiently large to encroach upon and rupture into openings that have an outlet from the body, (bronchial tubes in pulmonary tuberculosis, intestines in tabes mesenterica, etc.) This permits of invasion of all forms of pathogenic bacteria from without. We now no longer have a pure type of tuberculosis, but a mixed infection, the behavior of which depends upon the virulence of the germs and the resistance of the host. An effort is made to protect the structures against this invasion by laying down fibrous tissue more rapidly in the development of fibroblasts. This fibrous tissue may either by strong enough to wall off the invasion and stop its progress, or it may be so compact and deficient in blood supply as to undergo softening, liquifaction and breaking down. This breaking down is a saponaceous process, and nature, unwilling to abandon the fight still attempts to protect herself even here, by depositing lime salts in this saponaceous substance which may be sufficient in quantity to produce calcarius infiltration. This is her last effort to forestall the enemy.

After the disease has progressed thus far the gross pathology later is characterized according to the amount of structure involved, the nature of the involvement, and resistance of the individual. If there is but little resistance and much tissue involved the progress of the disease is rapid and the structures crumble and melt away before the advancing army of pathogenic germs. There is but little exudate, but little walling off, and the lungs become shot to pieces by multiple ulcerative areas. These areas merge into each other just as tubercles do, resulting in small cavities. Small cavities merging, or larger ones, thus continuing till death closes the scene. In tuberculosis of this type there are but little early physical signs except occasional rales, making it not only most malignant, but often latest diagnosed.

If there is resistance on the part of the host, with the germs only slightly aggressive, the walling off is more or less successful, the lung becomes infiltrated with fibrous tissue and exudative material, producing a chronic fibroid phthisis. If the germs are highly virulent and the resistance great, large quantities of exudative material are thrown out consisting of fibrin, white cells and a transudation of reds, producing a typical pneumonic process so that the disease often cannot be differentiated from lobar pneumonia, except by finding the tubercle bacilli, and from the history of the case.

On the other hand there may be large showers of tuberculous masses deposited all through either one or both lungs from which small tubercles develop and produce such a profound toxemia that death may ensue before caseation and confluence of the tubercles can take place. The macroscopic condition of the lungs is then characterized by small, hard shot-like bodies scattered throughout the structure. This is termed miliary tuberculosis. This type is not usually confined to the lungs but may affect all organs of terminal circulation.

Distinct lines of demarcation cannot always be drawn in these various forms by diagnostic methods as these forms not only merge into each other, but more than one or all of them may exist synchronously in different parts of the lungs of the same person.

428 Candler Bldg.

